# Evolution of social contacts patterns in France over the SARS-CoV-2 pandemic: results from the SocialCov survey

**DOI:** 10.1186/s12879-025-10611-4

**Published:** 2025-02-14

**Authors:** Flora Soussand, Armiya Youssouf Abdou, Marie Sanchez, Bich-Tram Huynh, Coralie Giese, Cécile Tran-Kiem, Guillaume Béraud, Didier Guillemot, Simon Cauchemez, Lulla Opatowski, Paolo Bosetti

**Affiliations:** 1 Mathematical Modelling of Infectious Diseases Unit, Institut Pasteur, Université Paris Cité, U1332 INSERM, UMR2000 CNRS, Paris, France; 2 Epidemiology and Modelling of Bacterial Escape to Antimicrobials, Institut Pasteur, Paris, France; 3 CESP, Anti-Infective Evasion and Pharmacoepidemiology Research Team, U1018, INSERM Université Paris-Saclay, UVSQ, Paris, France; 4 Data Management Core Facility, Institut Pasteur, Paris, France; 5 DGS, French Ministry for Health, Paris, France; 6 Vaccine and Infectious Disease Division, Fred Hutchinson Cancer Center, Seattle, WA USA; 7 Infectious Diseases Department, University Hospital of Orléans, LI²RSO, University of Orléans, Orléans, France

**Keywords:** Infectious disease dynamics, SARS-CoV-2, Social contacts, Age-structured contact matrices, Non-pharmaceutical interventions

## Abstract

**Background:**

Non-pharmaceutical measures such as lockdowns, curfews and place closures were implemented in France during 2020–2022 to reduce contacts in the population, to limit the spread of SARS-CoV-2 and reduce COVID-19 healthcare burden. Individuals also changed their behaviours as a response to the pandemic. Here, we present the results of the SocialCov survey that characterise the evolution of contacts in France between December 2020 and May 2022 to better understand the short and long term impact of these interventions on social mixing.

**Methods:**

A questionnaire was advertised over six independent communication campaigns through the governmental application TousAntiCovid between December 2020 and June 2022. Participants were asked to detail social contacts in the previous day, including contact age, location, duration and type (physical/conversational).

**Results:**

Over the six distinct campaigns, 44,396 individuals participated in the survey, declaring 300,735 contacts in total. The patterns of contacts strongly evolved over time, along with the progressive easing of national mitigation measures. The number of contacts in the French population increased from 5.3 contacts per day on average in December 2020 to 9.7 in May 2022. Mixing patterns were affected by age of participants, holidays and weekends. Healthcare workers declared 18.4 contacts on average during working days, roughly twice more than other workers. Reported risk perception changed throughout the two year period.

**Conclusions:**

Results provide a detailed picture of contact evolution over the years 2020–2022 in France. In addition to a major evolution of contact density over time, this study highlights strong heterogeneities in contact patterns according to age, employment and weekend/vacation periods. The contact matrices provided here can be used to inform age-stratified transmission models of respiratory pathogens in the context of implementation of multiple non-pharmaceutical measures.

**Supplementary Information:**

The online version contains supplementary material available at 10.1186/s12879-025-10611-4.

## Introduction

During the COVID-19 pandemic, faced with the risk of saturation of healthcare systems, many governments imposed stringent interventions to reduce the number of at-risk contacts in the population and control transmission. In France, multiple lockdowns, curfews and other mitigation measures (restaurants, bars, workplaces, university and school closure) were implemented in the first couple of years of the pandemic, strongly impacting the pattern and volume of social contacts in the population [1]. Other factors such as initial population acceptance of mitigation measures and individual protective behaviours followed by pandemic fatigue, COVID-19 vaccine implementation, may have interplayed with governmental measures to shape contact patterns during these unprecedented times.

We performed SocialCov, a large survey carried out in the French population during the SARS-CoV-2 pandemic to characterise and measure the evolution of mixing patterns. The first SocialCov campaign quantified the reduction of contacts during the first wave of SARS-CoV-2 infection in March 2020 following the implementation of the first lockdown in France [1]. It highlighted a dramatic reduction of contacts, on average by 70% during the lockdown period. However, contact patterns have strongly evolved afterwards. Here, we study the dynamics of contacts observed in France between December 2020 and May 2022, and analysing how age, occupation, and behaviour impacted contact patterns over time. We also investigate how mixing patterns changed during a period marked by different governmental measures and how the response of the population shaped contact patterns during a time characterised by the emergence of variants and the increased availability of vaccines. Finally, we provide contact matrices detailing mixing across ages in the French population over the study period. These matrices are key to characterise the drivers of respiratory diseases transmission by age under different types of restrictions.

## Methods

### SocialCov survey updates

SocialCov is an online survey monitoring contact patterns and behaviours among the French population. Briefly, the overall aim of the survey is to collect information on contact behaviours to understand how these contacts are distributed within the population. To this end, each participant is asked to report the number of contacts that occurred the previous day, and for each of these contacts, to indicate the age of the person and the place where the contact occurred. A first version of the survey was launched between 10 April and 28 April 2020, while France was under its national lockdown. The results for this time period were presented in a previous publication [1].

Following the first study, we revisited the questionnaire to better capture the changes in behaviour of the French population following the first lockdown. In order to inform more precisely their employment status, participants were asked to provide information on their job category, whether they were working in contact with sick people and/or whether their job involved being in contact with the public (e.g. drivers, shopkeepers etc.). We considered people whose work implied contact with sick people as healthcare workers. Additional contact information included at-home contacts with people who do not belong to the household, and, for contacts during leisure, some precision on location of contact (inside versus outside) and whether the contact was physical or not. If participants declared living with children, they were invited to complete a second specific child questionnaire for one of their children. Here, the term of children defines all participants under the age of 18. This second questionnaire was optional.

**Fig. 1:**
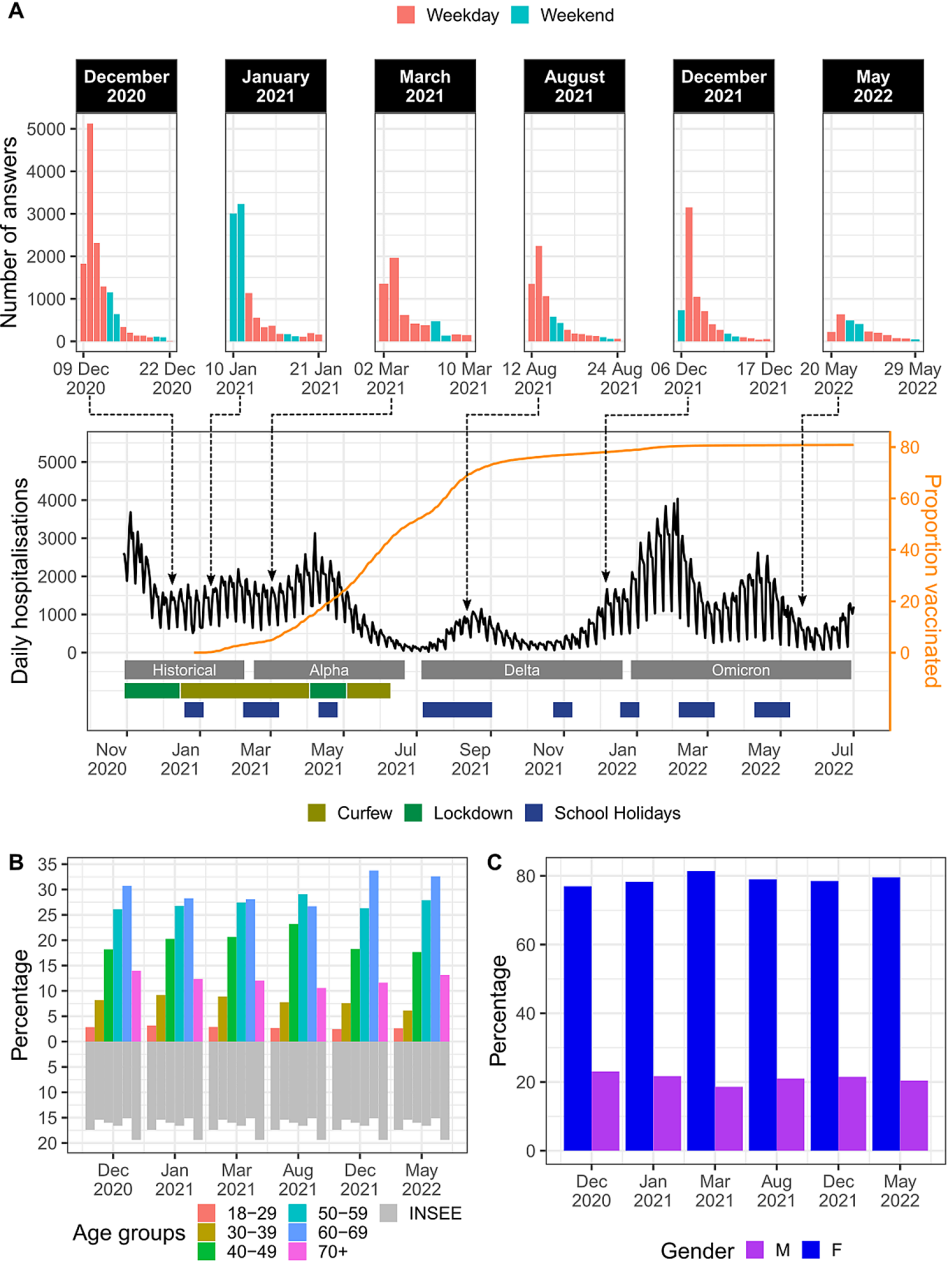
Recruitment campaigns and respondent population description. **(A)** Number of participants, epidemiological situations, enforced mitigation measures, vaccination coverage, and school holiday for each campaign. On the top panel, each plot represents the number of answers for each of the campaigns. For each day (x-axis), the bars represent the number of answers collected, with colours indicating whether the day when the contacts were declared (previous day) was a weekday or a weekend. The dashed lines represent the beginning date of a campaign. On the bottom panel, the black line represents the number of daily new hospitalizations (data from [31]) and the orange line represents the cumulative percentage of the French population that received at least one dose [32]. The horizontal bars below represent the periods corresponding to each dominant variant in France, the curfews and lockdowns as well as the periods when children from at least one zone were on holiday. **(B)** Comparison between the age distribution of respondents for each campaign (coloured bars) and the age distribution of the French population (grey bars) reported by the INSEE. **(C)** Distribution of respondents by gender for each recruitment campaign

As in the previous survey, contacts were defined as either a physical contact (such as a kiss or a handshake) or a close contact (such as face-to-face conversation at less than 1 m distance, see Text S1) [1]. As in [1] we use convenience sampling for recruiting participants. Survey communication was done exclusively through the news channel of the government app TousAntiCovid, inviting participants aged 18 years old and older to complete the questionnaire. TousAntiCovid is a contact tracing app developed by the French government to contain the spread of SARS-CoV-2 [2]. It also allows users to make travel documents and health certificates, such as records of vaccinations or negative test results, which may be used as sanitary passes, as well as to keep track of the current epidemiological situation. While widely encouraged, downloading the app was not obligatory. Six independent recruitment campaigns were initiated between 12 December 2020 and 1 June 2022. We defined the period of each recruitment campaign as the days included between the date of communication on TousAntiCovid to the first date with less than 50 responses collected in a single day (Fig. 1A). The duration of the campaigns ranged from 9 to 13 days.

Participants were asked to report the effective composition of their household on the previous day. In this study, we assumed that each participant had contact with all household members they reported while completing the survey.

### Statistical analyses

Adult participants were grouped according to six age groups: 18–29, 30–39, 40–49, 50–59, 60–69, ≥ 70 years old. For each campaign, participants whose declared number of contacts fell within the top 1% of the distribution were considered to have incorrectly filled out the survey and were removed from the analysis. Amongst the remaining participants we censored daily individual’s contacts at 50 contacts per day in order to match previously published contact surveys [3]. Individuals reporting more than 50 contacts in a day were considered as *hyperconnected*. Unless declared otherwise, this censoring was applied to all results. For *hyperconnected* individuals, the distribution of contacts in the different settings other than the household was maintained when censoring. Specifically, for each participant contacts with members of the household were kept as declared, while the remaining contacts (up to a total of 50) were redistributed according to the distribution over the different settings, i.e. respecting the proportion of contacts declared by the participant in the different other settings.

To generate a population representative of the age and gender distribution in France, sampling with replacement was performed from the pool of participants. For each campaign, we used data from the Institut national de la statistique et des études économiques (National Institute for Statistics and Economic Studies - INSEE) [4] to estimate the weights to apply to each gender and age group. Resampling was also performed to adjust the distribution of responses, with 5/7 of the contact survey answers sampled from participants reporting on weekdays and 2/7 from weekends, to ensure a more uniform representation across the week. Resampling was performed 500 times and relevant statistics were obtained, e.g. the mean and 95%CI (Confidence interval) for the mean number of contacts in the French population. When comparing child contacts with adult contacts, we similarly resampled the children population based on the distribution of age reported by INSEE [4].

We defined the two following levels of mask wearing:

- *Low*: not wearing a mask when outside home or wearing a mask irregularly.
- *High*: wearing a mask all or most of the time.

In the following, we use the term participant when presenting unweighted data and the term French population when presenting weighted data.

## Results

### Campaign contexts and participants characterization

After removing outliers, 44,396 participants’ answers were analysed over the six recruitment campaigns, with a maximum of 13,334 participants for the first campaign (December 2020) and a minimum of 2,500 for the sixth campaign (May 2022) (Fig. 1A). Because communication on TousAntiCovid did not always occur on the same weekday, the number of answers was not evenly distributed across weekdays and weekends between campaigns.

The epidemiological situation (COVID-19 hospital admission numbers or dominant variant), mitigation measures, vaccination coverage, and school term/holiday period strongly differed across the different recruitment campaigns (Fig. 1A). The first three campaigns were conducted within a short interval between December 2020 and March 2021. A curfew was in place in December 2020 starting at 8pm and ending at 6am. The curfew was intensified at the beginning of March (starting at 6pm) because of an increase in cases and hospital admissions caused by the Alpha variant. Vaccination was still in its early stage, with limited supplies; it was only available to people more than 60 years old and healthcare workers. The March 2021 campaign coincided with the school holidays in one of France’s three school zones. France is divided into these three zones to determine school holiday schedules across different regions. These zones are not geographically contiguous but are structured to evenly distribute the population among them. The fourth campaign was held in August 2021, during school summer break. At that time, vaccination was accessible for anyone aged 12 years old or more. By August 15 2021, 70% of the population had received their first dose [5]. During this period, severe restrictions (curfews or lockdown) had been eased, but a sanitary pass (i.e. certificate of recent vaccination or negative test) was necessary to access bars, restaurants, hospitals (except emergencies), retirement homes, airplanes, trains, and buses for long-distance travel, etc. The last two campaigns were conducted in December 2021 and in May 2022. Although the mask mandate for some specific public places and the requirement of a sanitary pass to access bars and restaurants were maintained during the December 2021 campaign, all of these measures were relaxed at the time of the May 2022 campaign.

The age distribution of participants remained similar across campaigns (Fig. 1B). People aged 50–69 years old (yo) were overrepresented in our survey corresponding to 56% of the participants (range across campaigns 54-61%) compared to 32% in the French population [4]. On the contrary, young adults, aged 18–39 yo, were underrepresented, representing 12% (range 9-13%) of the participants compared to 32% in the French population. Women were also overrepresented, accounting for 78% of participants (range: 77-81%) compared to 52% in the French population (Fig. 1C).

The recorded location of participants covered the entire metropolitan territory. The geographic distribution of participants in the different campaigns correlated well with the distribution of the population over the French departments, which represent one of the administrative divisions of France (metropolitan France is divided into 96 departments) (Figure S1). Correlation between the distribution of participants in each department and the distribution of inhabitants was high for each campaign and ranged from 0.89 in December 2021 to 0.74 in August 2021. For the campaign conducted during the summer holiday period in August 2021, the departments in the coastal and southern regions of France, which are typical holiday destinations, were overrepresented (Figure S2).

The distributions of participants according to the different socio-professional categories were constant over the different campaigns. Healthcare workers accounted for 11% of the participants while they represented 7% of the French population [6]. Participants with executive or higher intellectual professions were overrepresented, while those working in agriculture or manual occupations were generally underrepresented (Figure S3).

### Evolution of contacts in the French population over December 2020 - May 2022

**Fig. 2:**
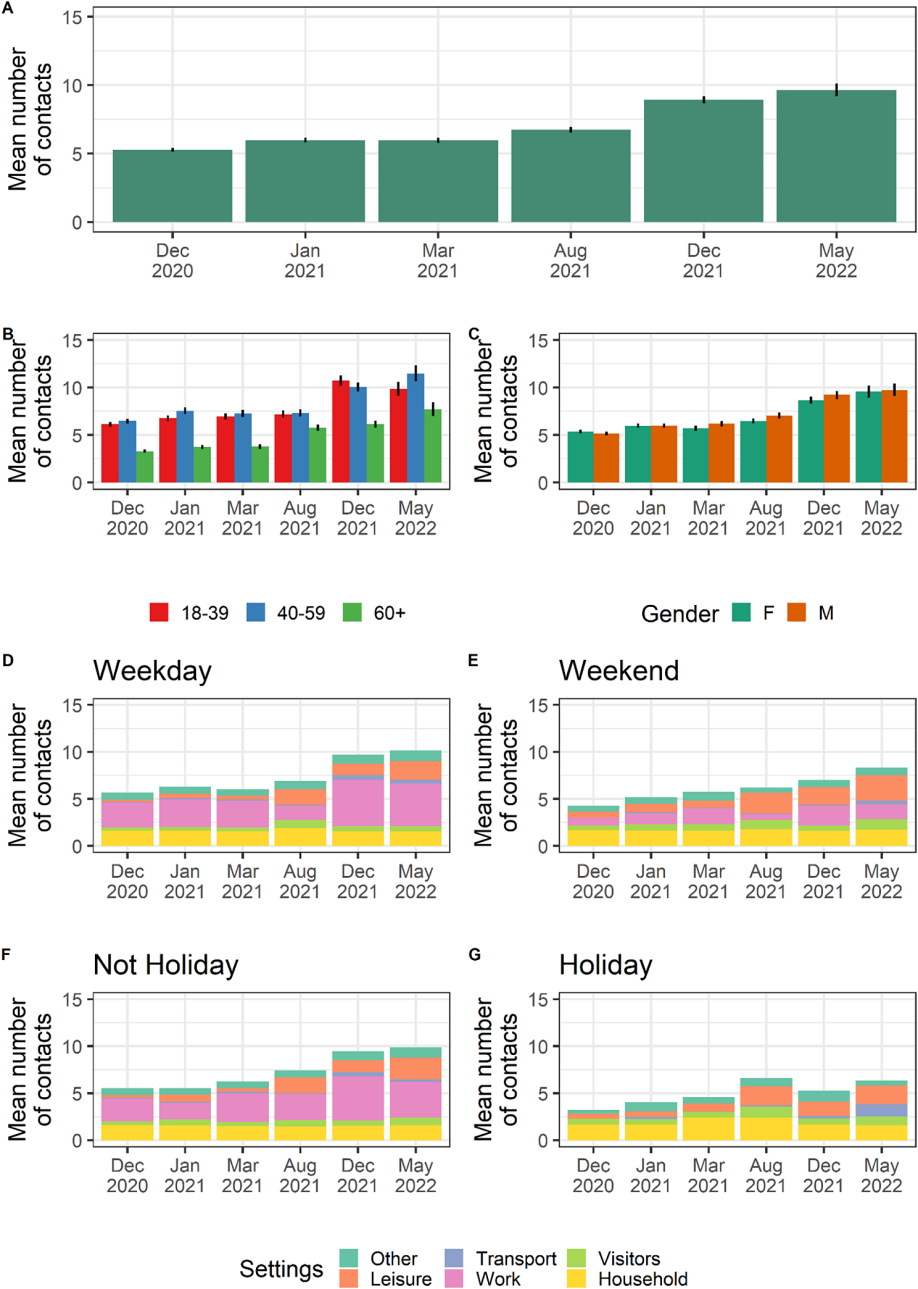
Evolution of contacts over time depending on age, gender and setting. **(A)** Average (and 95%CI, black line) number of contacts in the French population over the different recruitment campaigns. **(B)** Mean number of contacts (and 95%CI) by age group over the different campaigns. **(C)** Mean number of contacts by gender over the different campaigns. **(D)** Mean number of contacts by settings of contacts over the different campaigns during weekdays. Each color represents a different setting, and the height of each bar represents the mean number of contacts in this setting. **(E)** as (D) but for weekends. **(F)** as (D) but for people not on holiday. **(G)** as (D) but for people on holiday

A total of 300,735 contacts across 44,396 participants were reported. After censoring *hyperconnected* individuals (see Methods-section), our data consisted of 287,738 contacts. The average number of contacts in the French adult population increased from 5.3 (95%CI 5.1–5.4) daily contacts in December 2020 to 6.0 (95%CI 5.8–6.1) in January 2021, 6.0 (95%CI 5.8–6.2) in March 2021, 6.7 (95%CI 6.5-7.0) in August 2021, 8.9 (95%CI 8.7–9.2) in December 2021 and 9.7 (95%CI 9.2–10.1) daily contacts in May 2022 (Fig. 2A). This increasing pattern was observed across all age groups (Fig. 2B).

The average number of contacts in the population strongly varied with age (Fig. 2B). People over 60 yo declared 33–50% fewer contacts depending on the recruitment campaign than people aged 40–59 yo. The number of contacts did not differ between genders (Fig. 2C). Contact matrices by age are provided for each campaign at [7] and shown in Figure S4.

We found that contacts were less frequent during weekends compared with weekdays but that the extent of the frequency reduction varied across campaigns. The largest reduction was observed in December 2021 when the average number of contacts per person decreased from 9.7 (95%CI 9.4–10.0) on weekdays to 7.0 (95%CI 6.5–7.5) during weekends. In contrast, in March 2021, the average number of contacts per person did not show a clear difference varying from 6.0 (95%CI 6.3–5.8) during weekdays to 5.7 (95%CI 6.4–5.2) during weekends (Fig. 2D-E). Similarly, individuals on vacation reported having fewer daily contacts compared to those not on vacation. The largest reduction was observed in December 2021, where individuals not on vacation reported an average of 9.5 (95%CI 9.2–9.7) contacts per day, while those on vacation reported an average of 5.2 (95%CI 4.4–6.2) contacts per day, representing a 45% reduction. In August 2021, the reduction was only 10%, with individuals not on vacation reporting an average of 7.4 (95%CI 7.1–7.8) contacts per day compared to 6.7 (95%CI 6.4-7.0) for individuals on vacation (Fig. 2F-G).

Regarding the evolution of contacts in the different settings, on average, people ≥ 18 yo declared between 1.6 and 1.9 other members in their household during weekdays (Fig. 2D) in the different campaigns. For people declaring being on holidays, contacts in the household increased to 2.4 on average during both the campaigns of March 2021 and August 2021. The majority of reported contacts happened at work during weekdays (Fig. 2D) across all campaigns. Contacts at work increased over time, from an average 2.6 (95%CI 2.5–2.7) contacts per day during weekdays in December 2020, to 4.9 (95%CI 4.7–5.2) and 4.5 (95%CI 4.1-5.0) contacts per day in December 2021 and May 2022, respectively (Fig. 2D). Contacts during leisure time also increased over time. The mean number of contacts during leisure activities on weekdays was estimated at 0.3 (95%CI 0.2–0.3), 0.5 (95%CI 0.4–0.5), 0.4 (95%CI 0.4–0.5), 1.7 (95%CI 1.6–1.8), 1.2 (95%CI 1.1–1.3) and 2.0 (95%CI 1.7–2.3) in the different campaigns from December 2020 to May 2022.

### Contacts at work depending on occupation - focus on healthcare workers and people in contact with public

**Fig. 3:**
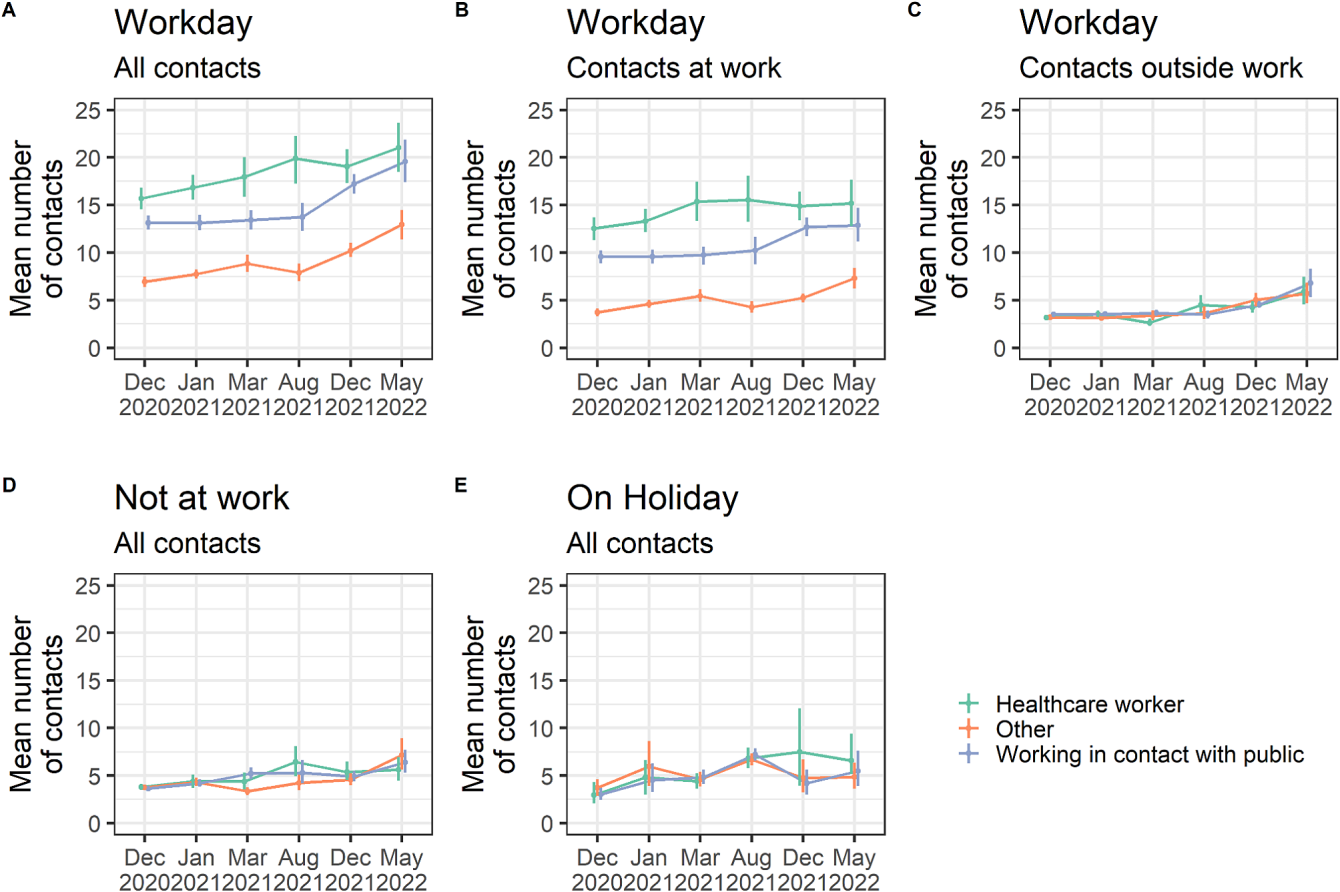
Comparison of contacts reported by healthcare workers, people working with the public and the rest of the working population. **(A)** Mean overall number of contacts, **(B)** mean number of contacts at work and **(C)** mean number of contacts outside of work for different workers’ categories during a workday. Colours represent the groups of workers: healthcare workers, people who work in contact with the public and other workers. For each campaign and workers’ group, we present the mean and 95% confidence interval (CI) of the number of contacts of the different categories. **(D)** Mean number of contacts for people who did not go to work on the previous day. **(E)** Mean number of contacts for people who declared being on holiday

We report much higher contacts for healthcare workers and people whose work imply contact with the public than the rest of the working population aged 30 to 60 years old (Fig. 3A). Interestingly, this difference was only observed for contacts at work on working days (Fig. 3B-C). Healthcare workers declared on average 18.4 contacts per day across campaigns (range 15.6–21.0) compared to 15.0 (range 13.1–19.6) daily contacts for people working with the public and 9.1 (range 6.9–13.0) daily contacts for the rest of the working population. In contrast, no difference was observed for contacts outside work during work days or for individuals who were not at work or on holidays (Fig. 3C-E).

### Evolution of at risk contacts and risk perception

**Fig. 4:**
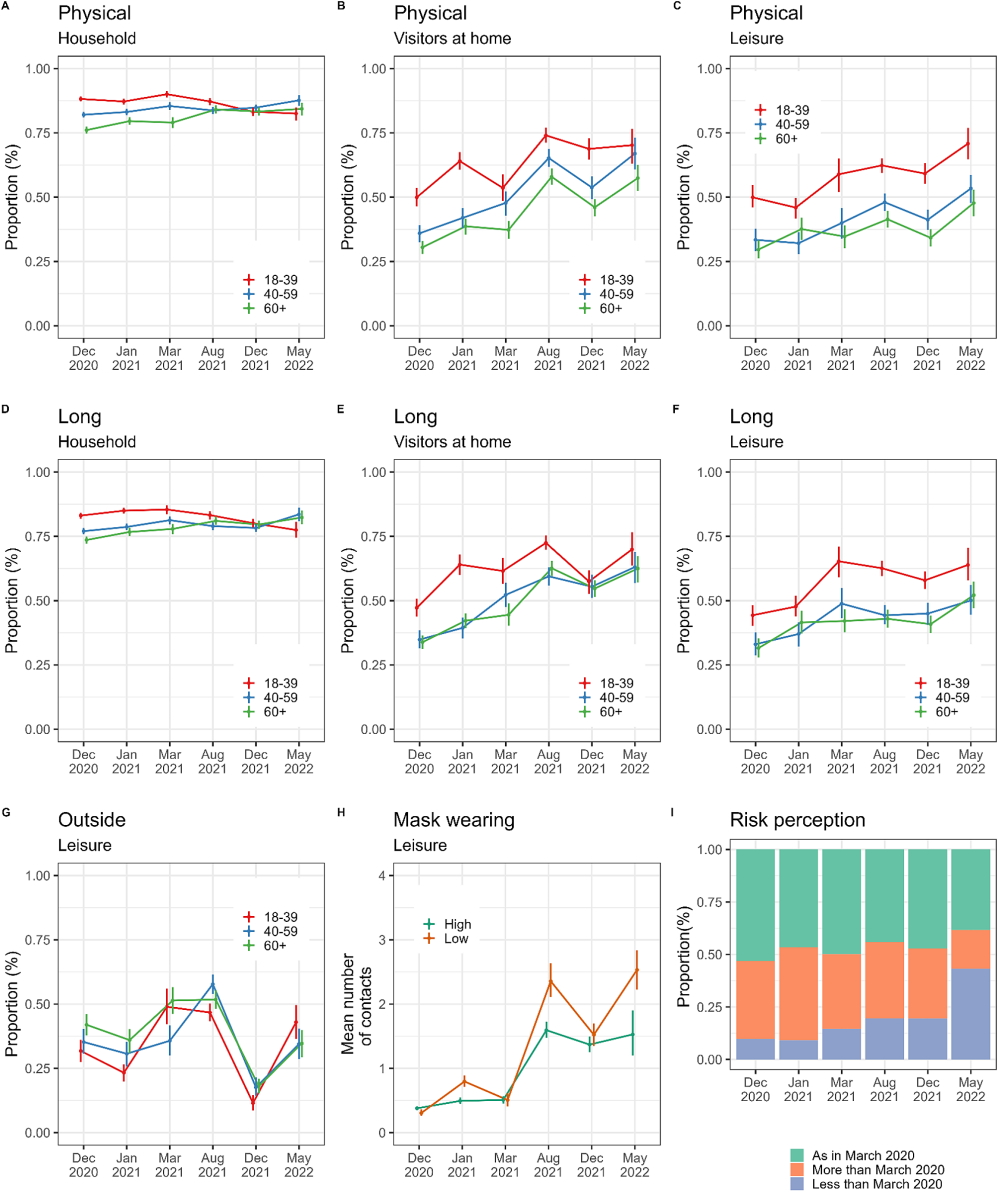
Type of contacts (physical or long), mean number of contacts associated with mask wearing behaviour and risk perception. **(A)** Proportion of physical contact for different age groups during each campaign at home with family members; **(B)** with visitors at home; or **(C)** in leisure settings. **(D)** Proportion of long contacts (> 30 min) for different age groups during each campaign at home with family members; **(E)** with visitors at home; or **(F)** in leisure settings. **(G)** Proportion of contacts outdoors during leisure for different age groups over the campaigns. **(H)** Mean number of contacts during leisure for each campaign for different mask wearing behaviour. **(I)** Proportion of people reporting a perception of risk similar/higher/lower as in March 2020 during each campaign

The percentage of physical or long-lasting contacts was approximately 80% among family members (under the assumption that all the people reported in the household were considered in close contact - see Methods-section). It was lower for contacts with visitors at home or during leisure activities, with a pattern that increased over time (Fig. 4). For instance, the proportion of physical contacts with visitors at home among people aged 18–39 yo increased from 50% (95%CI 47-54%) in December 2020 to 70% (95%CI 63-77%) in May 2022. A similar increasing pattern was observed for contacts during leisure activities. In particular, young adults were also more likely to have physical and long-lasting interactions than other adults. Young adults had 13% more physical contacts in May 2022 and up to 25% more in January 2021 compared to the elderly. When comparing the two December campaigns one year apart, it was observed that nearly 30% of leisure contacts for young adults occurred outdoors in December 2020. However, in December 2021, this percentage decreased to 11%.

Across the campaigns from August 2021 to May 2022, people who were less likely to wear masks had more social contacts than those who wore masks systematically (Fig. 4H). We estimate that, in May 2022, when the mask mandate had been lifted in France [8, 9], French people with lower mask wearing behaviour declared on average 2.5 (95%CI 2.2–2.9) contacts in leisure activities while other groups declared 1.5 (95%CI 1.2–1.9) on average. The proportion of people reporting a lower perceived risk associated with the epidemic with respect to March 2020 increased from 10% in December 2020 to 43% in May 2022 (Fig. 4I).

### Children’s contacts

The majority of participants that were asked to fill a questionnaire about one of their children did it (Range: 84%-73%, Figure S5). In total 9,289 children questionnaires were filled, representing an average of 1,367 questionnaires per campaign (range: 353–2707). Figure S6 shows the age distribution of children included in the study.

**Fig. 5:**
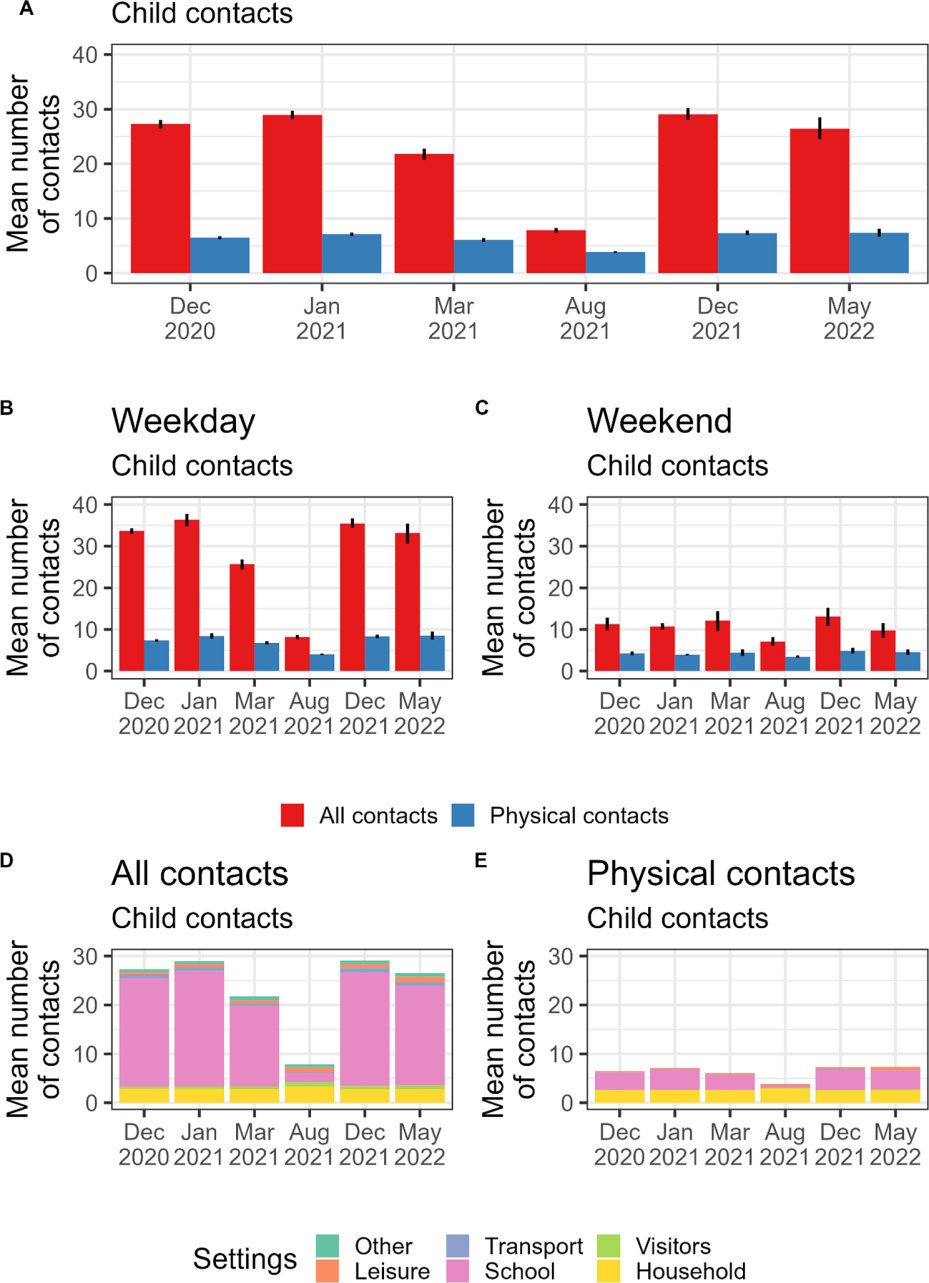
Contacts of the children. **(A)** Mean number (and 95%CI) of physical/ not physical contacts of the French children population during each campaign. (B) as (A) but for weekdays. **(C)** as (A) but for weekends. **(D)** Mean number of contacts of the French children population grouped by setting of contacts during each campaign. **(E)** as (D) but for physical contacts

The number of daily contacts reported for children was significantly higher than in the adult population. The mean number of contacts for children was in the range 26–29 except in March 2021 (approximately 22 contacts per day) and December 2021 (approximately 8 contacts per day), these two periods being associated with some period of school holidays (Fig. 5A). The mean number of physical contacts for children was much lower with respect to overall contacts, with about 4–7 physical contacts per day.

Selecting only the four campaigns not associated with school holidays, we found that children’s contacts were 68% (range over the campaigns 63-71%) lower during weekends than during weekdays (Fig. 5B-C). During those campaigns between 76% and 82% of children’s contacts happened at school (Fig. 5D).

The number of household contacts for children was stable at 2.8–2.9 daily contacts on average, except in August 2021, when it increased to 3.3 daily contacts. In the household, 91% (range 89-93%) of children’s contacts were physical while that proportion dropped to 17% (range 16-19%) at school (Fig. 5B).

## Discussion

The COVID-19 pandemic and the measures implemented by governments to mitigate its impact heavily modified social contacts at population-level. Because respiratory viruses such as SARS-CoV-2 typically spread through close contacts, quantifying these modifications is crucial to evaluate the impact of those measures [10]. In the present work, we analysed the evolution of contacts observed in France between December 2020 and May 2022, a period characterised by a gradual easing of restrictions until their complete removal, highlighting strong heterogeneities of contacts depending on age, profession, and activity.

With an overall 83% increase in the average number of contacts, we found that behaviours evolved throughout the study period. In December 2020, when a curfew was in place in France, 5.3 (95%CI 5.1–5.4) contacts per day were reported on average in the French population, a significantly higher estimate than the 3.3 contacts per day estimated in April 2020 during the first lockdown [1]. Throughout the different campaigns, this number of contacts kept increasing, reaching values similar to those measured prior to the pandemic. We report 9.7 (95%CI 9.2–10.1) daily contacts on average in May 2022 compared to 9.5 in the pre-pandemic COMES-F study [11]. This is also consistent with data from Google mobility trends showing that, in May 2022, attendance of settings such as workplaces, transit stations, etc. was comparable to pre-pandemic periods [12]. This reported progressive increase in contact rates could be the result of the combination of relaxing NPIs, reduction of perceived risk (partly due to widespread vaccination) and possible fatigue in limiting social interactions. Interestingly, other studies from 2022 [13] reported a long lasting change in the mixing patterns, with a persistently lower frequency of contacts compared to pre-pandemic POLYMOD surveys. The origin of such discrepancy is unclear, and further studies will be important to determine whether such decreases are maintained over time, potentially changing the epidemiology of other pathogens.

During the campaigns of August 2021, adults between 18 yo and 60 yo reported an unexpectedly low number of contacts despite the important easing of restrictions at the time. The intensity of contacts was only 12% higher than the one observed in March 2021 under much more restrictive measures in place (e.g. a curfew at 6 pm). This is likely because a substantial proportion of the French population was on vacation at the time. Along with the increase in the vaccination coverage, this decrease in contact rates during holidays probably contributed, to reverse the sharp rise in cases that occurred during the emergence of the highly transmissible Delta variant in France [14].

Consistent with research conducted in high-income countries [11, 15, 16], we report a strong heterogeneity of contacts across age groups. During the May 2022 campaign, we estimated that people aged 60 yo had on average 33% fewer contacts than adults aged 40–59 yo. This is comparable with previous pre-pandemic surveys: in the French pre-pandemic contact survey COMES-F [11], people aged > 65 yo) reported 24% fewer contacts than people aged 45–64 yo. In the POLYMOD survey [16] people aged > 70 yo have 44% fewer contacts compared to people aged between 50 and 59 yo.

Our results suggest that healthcare workers and workers in contact with the public have significantly more social contacts than the rest of the population. We also observed a greater number of contacts among individuals who reported wearing masks less systematically. This aligns with the observation, as noted in [17], that people who perceived a low level of severity associated with a possible SARS-CoV-2 infection reported having more daily contacts compared to those with a high perceived severity. The heterogeneity in contact patterns and existence of subgroups with a significantly higher number of contacts is an important feature of the population that can have epidemiological consequences. Some modelling studies have shown that such heterogeneity can lower the immunity threshold required to achieve herd immunity in the population [18]. A few studies investigated contact networks in health care settings, disentangling the part of contacts that occurred between healthcare workers or between an healthcare worker and a patient [19–22]. It is important to stress that even if the number of contacts of healthcare workers is higher, they may involve more precaution than contacts from the general population, leading to a reduced transmission risk per contact.

Significant heterogeneities were identified not only in the contact frequency but also in the intensity of those contacts, as measured by the type of contacts (physical or not) or their duration. Although exact duration of contact was not available here, we found that young adults had a higher proportion of physical and long-lasting contacts than the rest of the adult population in leisure settings. Contact intensity has been suggested to be an important driver for the spread of infectious diseases given prolonged and physical contacts are more likely to result in transmission [23].

Overall, estimated numbers of contacts for children were very high during school term, with an average number of contacts ranging 26–29. These values are much higher than values estimated for adults. These estimates are also higher than the ones reported in the French CoMix survey [3, 24], which reported in February 2021, 10.4 daily contacts in children. The two studies differed in their recruitment modalities; CoMix was based on quota sampling by opposition to convenience sampling in SocialCov; and in CoMix, the same participant cohort was engaged in answering for the different waves, which has been suggested to lead to some reporting fatigue. However, the reasons for such differences are unclear, especially as the definition of a contact was the same in the two studies.

The frequencies of children’s contacts evolved over the different campaigns, from about 27 daily contacts during school terms to 8 during summer holidays in August 2021. These variations support the generalised idea that school closures can help diminish the contacts between children, with possible effect in slowing down the progression of infectious disease in this population [25–27]. However, in the specific context of SARS-CoV-2, children and adolescents were shown to be less susceptible to infection than adults, especially to the Wuhan historical variant at the initial phases of the pandemic [28]. Such property reduces the global effect of interventions meant to reduce children contacts, such as school closures. School closure can also modify the global distribution of contacts in the community, possibly changing inter-generational contacts, therefore altering the risk in other age groups. In the future more epidemiological including behavioural studies should be done in specific community settings such as schools or households.

Our results should be interpreted in the light of the following limitations. First, participants completed the questionnaire online, self-reporting their contacts anonymously, with no possibility of validation by investigators. To account for potential errors in completing the questionnaire, we removed the top 1% of the distribution.

Second, because recruitment was based on convenience sampling following communication campaigns on the TousAntiCovid governmental application, our survey population was not representative of the French population. This might explain some differences with the COMES-F study where participants were recruited according to quota for age, gender, days of the week and school holidays and were sent a diary to complete. Individuals aged 40–69 yo were overrepresented in our sample, and as a consequence, children’s age distribution was also skewed towards older age groups, as illustrated in figure S6. Similarly, higher intellectual professions were over-represented in the responding population. To overcome this issue, synthetic populations more representative of age and gender distribution in France were reconstructed for each campaign, using sampling with replacement in the SocialCov participants population. Yet, because of limited sample size, resampling to match work categories distribution in France was not possible. As a consequence, people declaring being employed as higher intellectual professions were also over-represented in our synthetic population (Figure S3). Because the different sampling designs strongly differ in terms of investment and cost, it would be critical to investigate how choice in study design actually impacts the estimation of contact matrices over time. Future studies comparing different study designs should be implemented to provide such evaluation.

Second, the questionnaire did not include specific questions related to casual at-home contacts: in our data, all declared household members were considered as systematic at-home contacts of the participant assuming uniform mixing across individuals in the household. This hypothesis might not be true on all days: heterogeneity in contact mixing was described in households depending on the individual’s status (age, parents, siblings) [29, 30]. Dedicated surveys should be set up in the future to assess more precisely patterns of contact across ages and between categories within households.

## Conclusions

We highlight strong heterogeneity across ages, categories and slow increase of contact frequency over the period December 2020 and May 2022. The presented data and matrices can be used to inform mathematical age-structured models of respiratory pathogens transmission and help evaluate the impact of non-pharmaceutical measures in future pandemics.

## Electronic supplementary material

Below is the link to the electronic supplementary material.

Supplementary Material 1

## Data Availability

Data on contact matrices are provided via a Zenodo link [7].
