# Supplementary material for: Evolution of social contacts patterns in France over the SARS-CoV-2 pandemic: results from the SocialCov survey
Source: BMC Infect Dis. 2025 Feb 14;25:224. doi: 10.1186/s12879-025-10611-4 (PMC11829358; doi:10.1186/s12879-025-10611-4)
Supplement: Supplementary file 1 — Supplementary Material 1 [file 12879_2025_10611_MOESM1_ESM.pdf]

Supplementary material: Evolution of social contacts patterns in France over the SARS-CoV-2 pandemic: results from the SocialCov survey

Text S1. Definition of contacts to be declared in the survey:

The questionnaire was preceded by the following text defining what types of contact should be reported in the survey.

French version (survey): Nous allons vous interroger dans la suite de l'enquête sur vos contacts. Nous vous prions d'indiquer dans cette enquête toutes les personnes avec lesquelles vous avez été en contact.
Quelles personnes déclarer ?
- Les personnes avec qui vous avez eu un contact oral : vous avez parlé avec quelqu'un en sa présence physique et à une distance inférieure de 1 mètre.
- Les personnes avec qui vous avez eu un contact physique : vous avez touché une autre personne (se donner ou serrer la main, s'embrasser, se donner l'accolade, frôler ou bousculer quelqu'un, s'échanger un objet ou autre ...).
Sont exclues les personnes avec qui :
- vous avez eu un contact par téléphone ou internet
- vous avez eu un contact ayant donné lieu à une discussion non rapprochée (plus de 1 mètre).
Si vous avez été en contact plusieurs fois avec une même personne dans une journée, même dans des lieux différents, nous vous demandons de ne la déclarer qu'une fois.

English version (Translation): In the rest of the survey, we will be asking you about your contacts. Please indicate in the survey all the people with whom you have been in contact.
Who should I tell?
- People with whom you had verbal contact: you spoke with someone in their physical presence and at a distance of less than 1 meter.
- People with whom you have had physical contact: you have touched another person (giving or shaking hands, kissing, hugging, brushing or jostling someone, exchanging an object, etc.).

This does not include people with whom :
- you have had contact by telephone or internet
- you have had contact that resulted in a non-close discussion (more than 1 meter).
If you have been in contact with the same person several times in one day, even in different places, we ask you to declare them only once.

Additional results

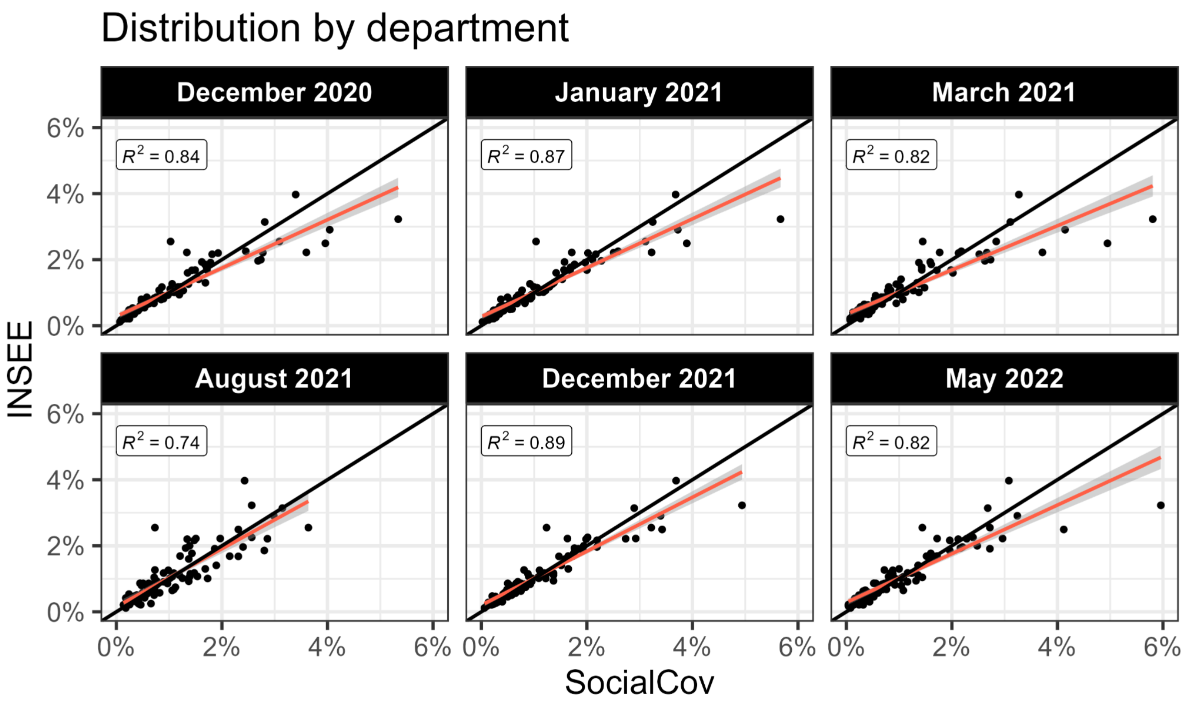

Figure S1: Scatter plot showing the relationship between the population distribution across departments in France (y-axis), based on national census data (INSEE), and the distribution of participants in each department for recruitment campaigns (x-axis).

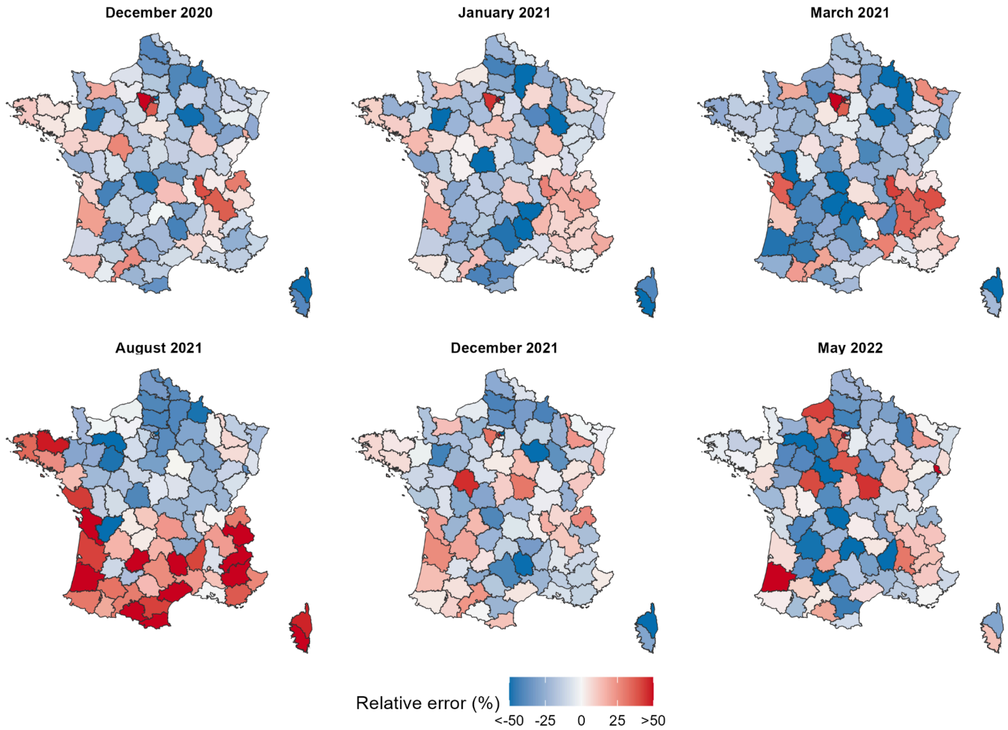

Figure S2: Relative error between the population distributions for each department and the distribution of participants for each wave of the campaign. The relative error for each department i is computed using the formula: relative_error_i(c) = (dist_SocialCov_i(c) - dist_INSEE_i) / dist_INSEE_i, where dist_SocialCov_i(c) and dist_INSEE_i represent the distribution of participants during recruitment campaign c and the population distribution recorded by INSEE for each department, respectively.

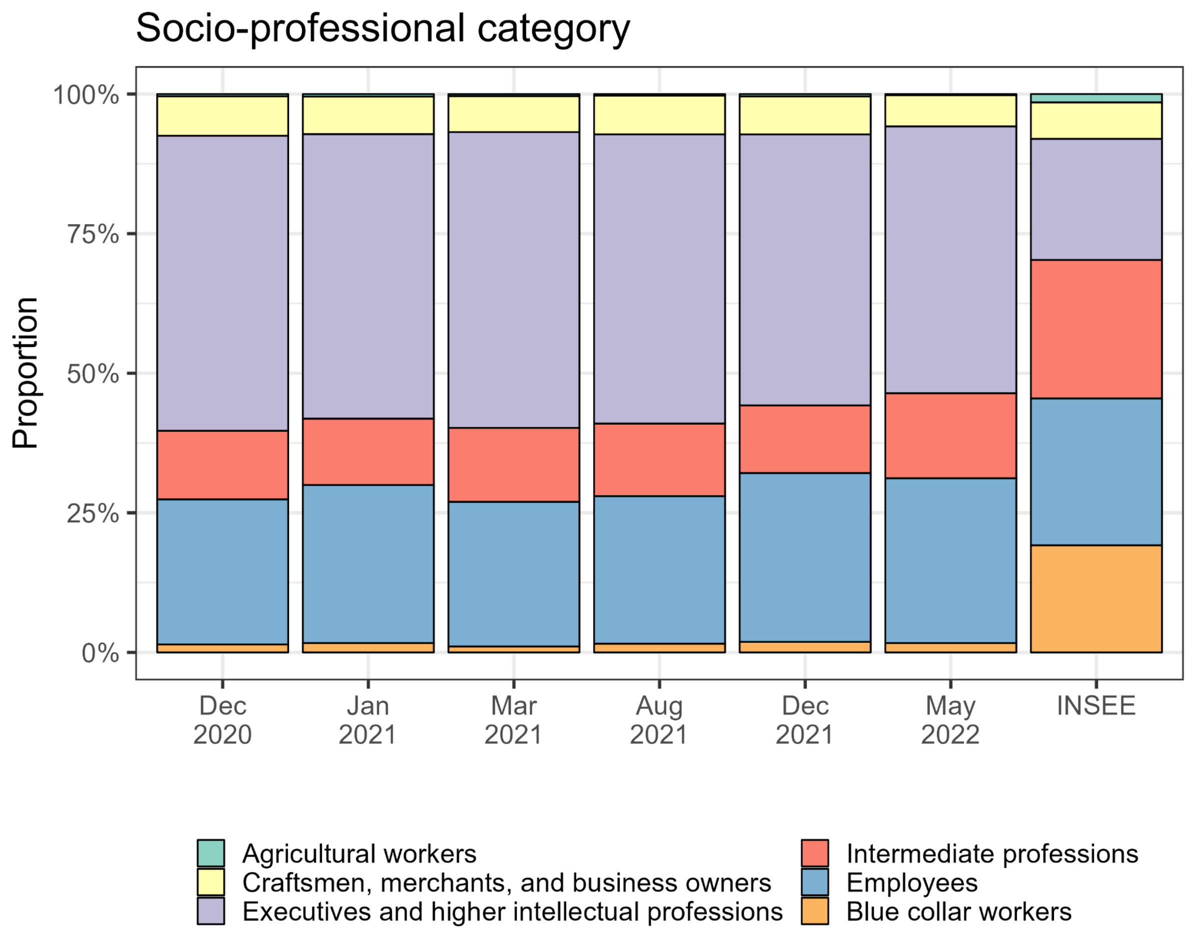

Figure S3: Proportion of participants in the different socio-professional categories.

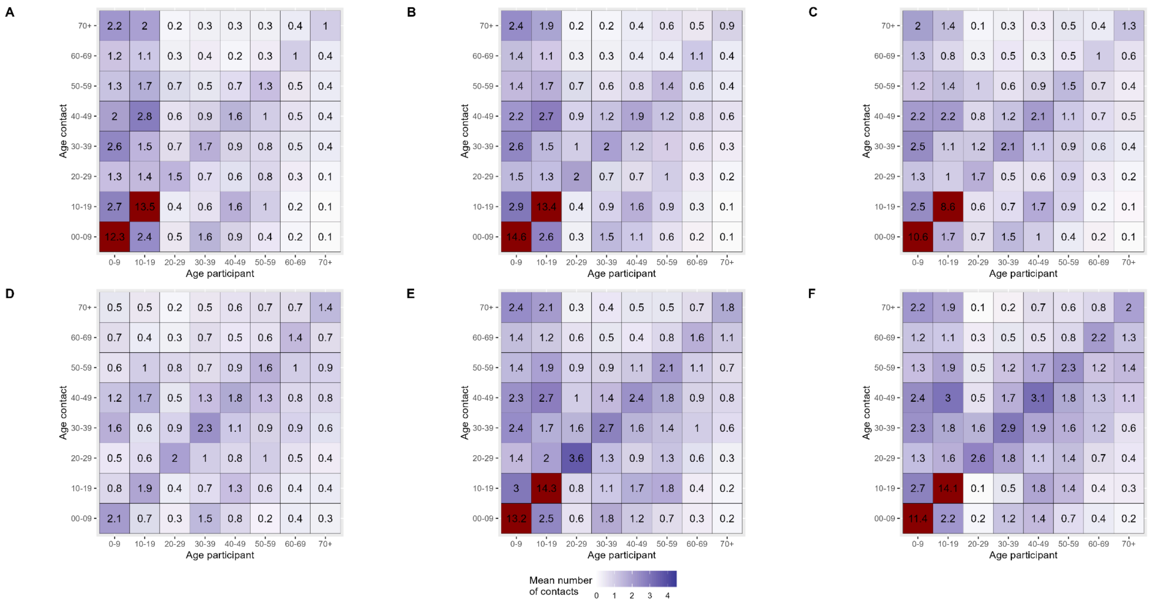

Figure S4: Contact matrices for all the different campaigns of recruitment adjusted for socio demographics covariates. A for December 2020 campaign. **B** for January 2021 campaign. **C** for March 2021 campaign. **D** for August 2021 campaign. **E** for December 2021. **F** for May 2022 campaign.

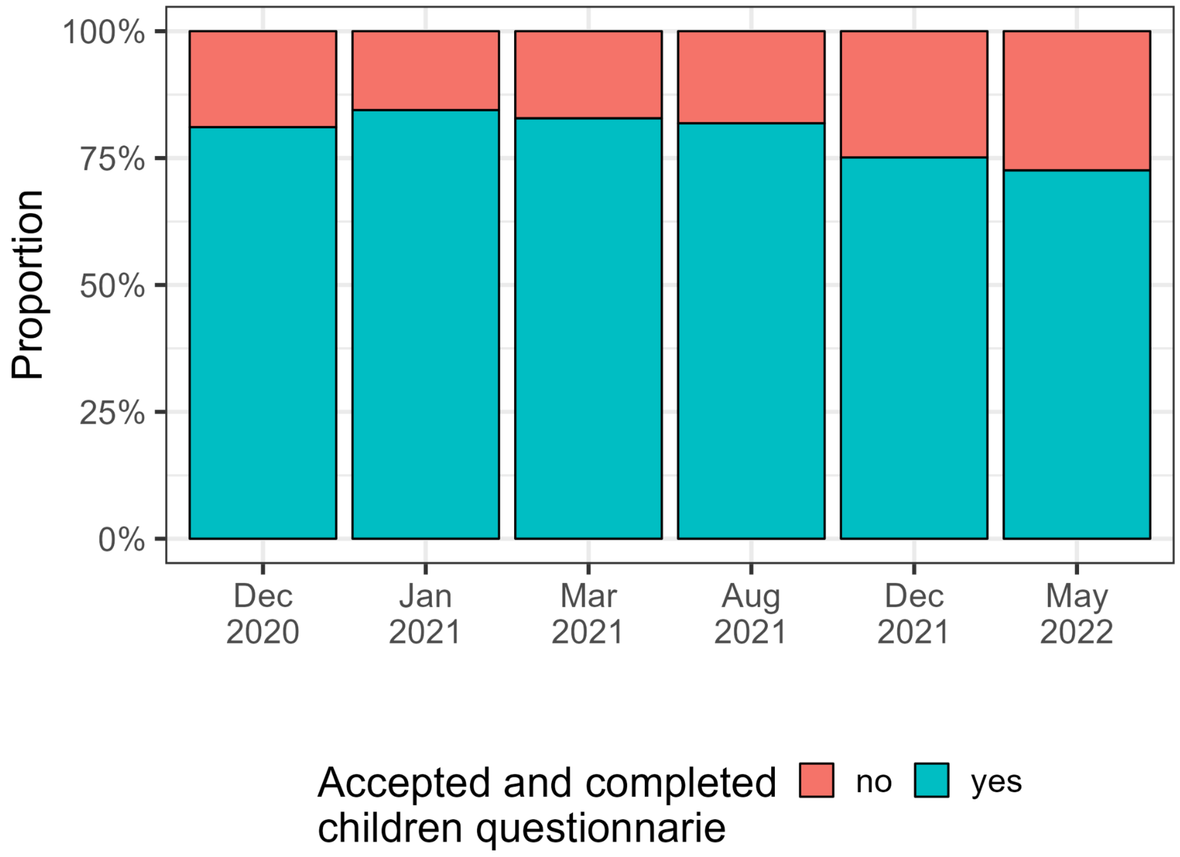

Figure S5: Proportion of participants with children in the household who accepted and completed the questionnaire for their children.

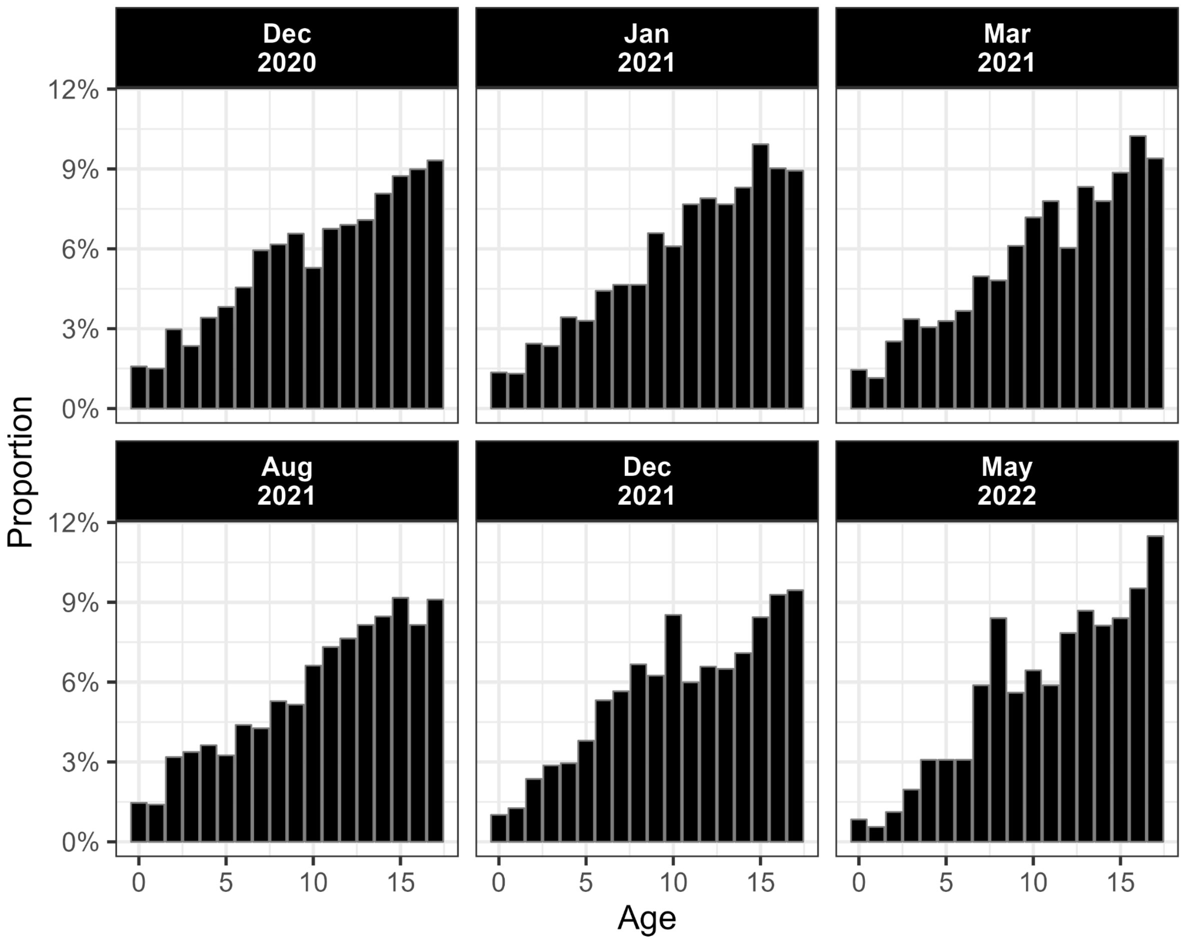

Figure S6: Age distribution of children during the different recruitment campaigns.
